# Phase 2 study of azacitidine plus pembrolizumab as second-line treatment in patients with locally advanced or metastatic pancreatic ductal adenocarcinoma

**DOI:** 10.1093/oncolo/oyag091

**Published:** 2026-03-17

**Authors:** Rachael A Safyan, Ruth A White, Tamas A Gonda, Shing M Lee, Jiying Han, Nadine Kuriakose, Naomi K Yamamoto, Sita Kugel, Jacob K Jamison, Gulam A Manji, Gary J Schwartz, Paul E Oberstein, Susan E Bates

**Affiliations:** University of Washington School of Medicine, Seattle, WA, 98195, United States; Fred Hutchinson Cancer Center, Seattle, WA, 98109, United States; Laura and Isaac Perlmutter Cancer Center, NYU Langone Health, New York, NY, 10016, United States; Laura and Isaac Perlmutter Cancer Center, NYU Langone Health, New York, NY, 10016, United States; Columbia University Mailman School of Public Health, New York, NY, 10032, United States; Columbia University Mailman School of Public Health, New York, NY, 10032, United States; Herbert Irving Comprehensive Cancer Center, Columbia University Irving Medical Center, New York, NY, 10032, United States; Department of Medicine, James J. Peters Bronx VA Medical Center, Bronx, NY, 10468, United States; Fred Hutchinson Cancer Center, Seattle, WA, 98109, United States; Fred Hutchinson Cancer Center, Seattle, WA, 98109, United States; Weill Medical College, Cornell University, New York, NY, 10065, United States; Herbert Irving Comprehensive Cancer Center, Columbia University Irving Medical Center, New York, NY, 10032, United States; Case Comprehensive Cancer Center, Cleveland, OH, 44106, United States; Laura and Isaac Perlmutter Cancer Center, NYU Langone Health, New York, NY, 10016, United States; Herbert Irving Comprehensive Cancer Center, Columbia University Irving Medical Center, New York, NY, 10032, United States; Department of Medicine, James J. Peters Bronx VA Medical Center, Bronx, NY, 10468, United States

**Keywords:** pancreatic cancer, azacitidine, pembrolizumab, tumor immune microenvironment

## Abstract

**Background:**

Epigenetic regulators represent a novel strategy to modulate the tumor immune microenvironment in pancreatic ductal adenocarcinoma (PDAC). In preclinical models, DNA hypomethylating agents enhance cytotoxic T-cell infiltration, synergize with PD-1 blockade, and improve survival when combined with immune checkpoint blockade. This single-institution, phase II study evaluated the safety, efficacy, and biomarkers of azacitidine plus pembrolizumab in patients with previously treated PDAC.

**Methods:**

Patients with locally advanced or metastatic PDAC after one prior regimen received 50 mg/m^2^ subcutaneous azacitidine on days 1-5 of a 28-day cycle, starting week 1, and pembrolizumab 200 mg intravenously every 3 weeks starting week 3. Baseline and on-treatment blood and tumor was collected for exploratory biomarker analysis.

**Results:**

Thirty-six patients enrolled between October 2017 and September 2021 (median age: 62.5 years); 34 were evaluable for safety; 31 for efficacy. Treatment was generally well-tolerated, with Grade 1-2 fatigue and diarrhea most common AEs. Three patients (9.7%) had a partial response, and the disease control rate was 35.5%. Median progression-free and overall survival was 1.51 and 4.83 months, respectively. Exploratory analysis suggested higher baseline CD8^+^ T cells and lower tumor Ki-67 was associated with response, whereas low baseline CD8+ T cell and Granzyme B infiltration correlated with higher exponential tumor growth rate. PD-L1 and CD68 expression were not predictive of benefit.

**Conclusion:**

Azacitidine plus pembrolizumab demonstrated limited clinical activity in second line, locally advanced or metastatic PDAC. Biomarker analysis suggests higher baseline CD8^+^ T-cell infiltration and lower proliferative index may identify patients more likely to benefit. Clinical trial registration number: NCT03264404.

## Lessons learned

- Azacitidine plus pembrolizumab had a manageable safety profile but showed limited efficacy compared to historical controls in second line locally advanced or metastatic PDAC.
- Baseline PD-L1 expression was not associated with treatment response.
- Higher tumor-infiltrating CD8^+^ T cells and lower Ki-67 proliferation index correlated with reduced tumor growth rate and may serve as biomarkers of response to epigenetic priming plus immune checkpoint blockade.

**Table:** 

**TRIAL INFORMATION**	
**Disease**	Pancreatic ductal adenocarcinoma
**Stage of disease/treatment**	Locally advanced unresectable and metastatic/azacitidine plus pembrolizumab
**Prior therapy**	1 prior regimen
**Type of study**	Phase II
**Primary endpoints**	Progression-free survival per RECIST 1.1
**Secondary endpoints**	Overall response rate, duration of response, overall survival, and safety
**Additional details of endpoints or study design** **Trial design and treatment** This was a single-arm, single-center, open-label, phase II trial to evaluate the safety and efficacy of azacitidine in combination with pembrolizumab in patients with previously treated, locally advanced unresectable or metastatic PDAC. Archival tumor or a pre-treatment biopsy was required. An on-treatment biopsy from the primary tumor or a metastatic site was required at week 8 for correlative biomarker analysis. Azacitidine 50 mg/m^2^ was administered subcutaneously on days 1-5 of each 28-day cycle, beginning on week 1, day 1. Pembrolizumab 200 mg was administered intravenously every 3 weeks, starting on week 3. A total of 34 patients were evaluable for safety—defined as those who received at least one dose of azacitidine. Three patients did not proceed to receive pembrolizumab, leaving 31 who received both agents. **Treatment duration** Treatment could continue for up to 2 years (35 cycles of pembrolizumab maximum) or until confirmed disease progression per RECIST 1.1, development of unacceptable toxicity, intercurrent illness, withdrawal of consent, or investigator decision to discontinue treatment. **Dose modifications and safety assessments** Dose adjustments of azacitidine were permitted for the management of adverse events (AEs). Pembrolizumab dose modifications were not allowed, although treatment delays or interruptions for either drug due to toxicity were permitted. All patients were evaluable for toxicity from the first dose of azacitidine. There was a run-in of evaluable 6 patients to assess safety. Evaluable patients were defined as those who received at least one dose of pembrolizumab following the initial week of azacitidine. Dose-limiting toxicity (DLT) was defined as a grade ≥3 AE occurring within the first 6 weeks of treatment and attributed to either study drug, or any AE necessitating permanent discontinuation of azacitidine or pembrolizumab. AEs were graded using the National Cancer Institute (NCI) CTCAE v4.0. Safety assessments included clinical laboratory evaluations (complete blood count, complete metabolic panel), physical examinations, and systematic AE documentation. Serious adverse events (SAEs) were defined as any unfavorable incident leading to fatal consequences, a life-threatening situation, necessitating inpatient hospitalization, extending an ongoing hospitalization, resulting in enduring or significant impairment contributing to a congenital anomaly or birth defect, or otherwise deemed medically imperative. **Inclusion criteria** Histologically or cytologically confirmed PDAC. ECOG performance status 0-1 within 3 days prior to the first study dose Adequate organ function within 7 days prior to the first study dose. Predicted life expectancy >3 months. Measurable disease per RECIST 1.1. Age ≥18 years at time of consent. Radiographic disease progression or intolerance to a prior line of systemic therapy, which must have included 5-FU (or capecitabine)- or gemcitabine. Patients with recurrence >6 months after completing neoadjuvant or adjuvant chemotherapy for localized disease were eligible only if additional chemotherapy for advanced disease had been administered. Resolution of all prior therapy-related toxicities (except alopecia and fatigue) to ≤ grade 1 or baseline per (NCI-CTCAE v4.0); chronic toxicities not expected to resolve (eg, platinum-induced neuropathy), were allowed. **Exclusion criteria** Chemotherapy or radiotherapy within 14 days of first study dose. Prior solid organ or hematologic transplant. >10% weight loss within 2 months prior to first study dose. Active autoimmune disease requiring systemic therapy within the past 2 years. Known immunodeficiency, chronic steroids at a dose >10 mg/day of prednisone equivalent, or any other form of immunosuppressive therapy within 7 days of study start. Second malignancy diagnosed within 2 years, excluding curatively treated basal/squamous cell carcinoma of the skin or resected *in situ* breast cancer. Interstitial lung disease or active, non-infectious pneumonitis. Active infection requiring systemic therapy. Clinically significant ascites at baseline (requiring paracentesis or moderate by imaging); minimal ascites permitted. Any condition or comorbidity that, in the investigator’s judgment, could interfere with study participation or outcomes (eg, dialysis). Psychiatric or substance use disorders that would interfere with protocol compliance. Prior treatment with immune checkpoint inhibitors (anti-PD-1, anti-PD-L1, or anti-PD-L2 agents). Known human immunodeficiency virus (HIV) infection, or active/chronic Hepatitis B or C infection.	

**Table:** 

**DRUG INFORMATION**	
**Generic/working name**	Azacitidine/Vidaza
**Company name**	Bristol Myers Squibb
**Drug type**	Pyrimidine nucleoside analog
**Drug class**	Demethylation agent
**Dose**	50 mg/m^2^
**Route**	Subcutaneous
**Schedule of administration**	Days 1-5 every 28 days

**Table:** 

**DRUG INFORMATION**	
**Generic/working name**	Pembrolizumab/Keytruda
**Company name**	Merck
**Drug type**	PD-1 inhibitor
**Drug class**	Cancer immunotherapy
**Dose**	200 mg
**Route**	Intravenous
**Schedule of administration**	Day 1 every 21 days beginning week 3

**Table:** 

**PATIENT CHARACTERISTICS**	
**Number of patients, male**	25 (74%)
**Number of patients, female**	9 (26%)
**Stage**	Locally advanced, 1; metastatic, 33
**Age: median (range)**	62.5 (48, 83)
**Number of prior systemic therapies: median (range)**	1
**Performance status: ECOG 0 or 1**	34 (100%)
**Performance status: ECOG 2 or above**	0
**Cancer types or histologic subtypes**	Ductal adenocarcinoma: 34

Among the 34 patients, the median age was 62.5 years (range: 48-83). Most patients (33 of 34; 97.1%) had metastatic disease and liver involvement was the most common site (66.7%) followed by lung (35.4%) and peritoneum (33.3%). Fifteen patients (44.1%) had undergone prior surgery on the primary tumor. The median CA 19-9 at study enrollment was 647. All patients received one prior line of chemotherapy, with 22 patients (64.7%) having received a 5-FU-based regimen.

## Primary assessment method

**Figure 1. | Patient flowchart.**
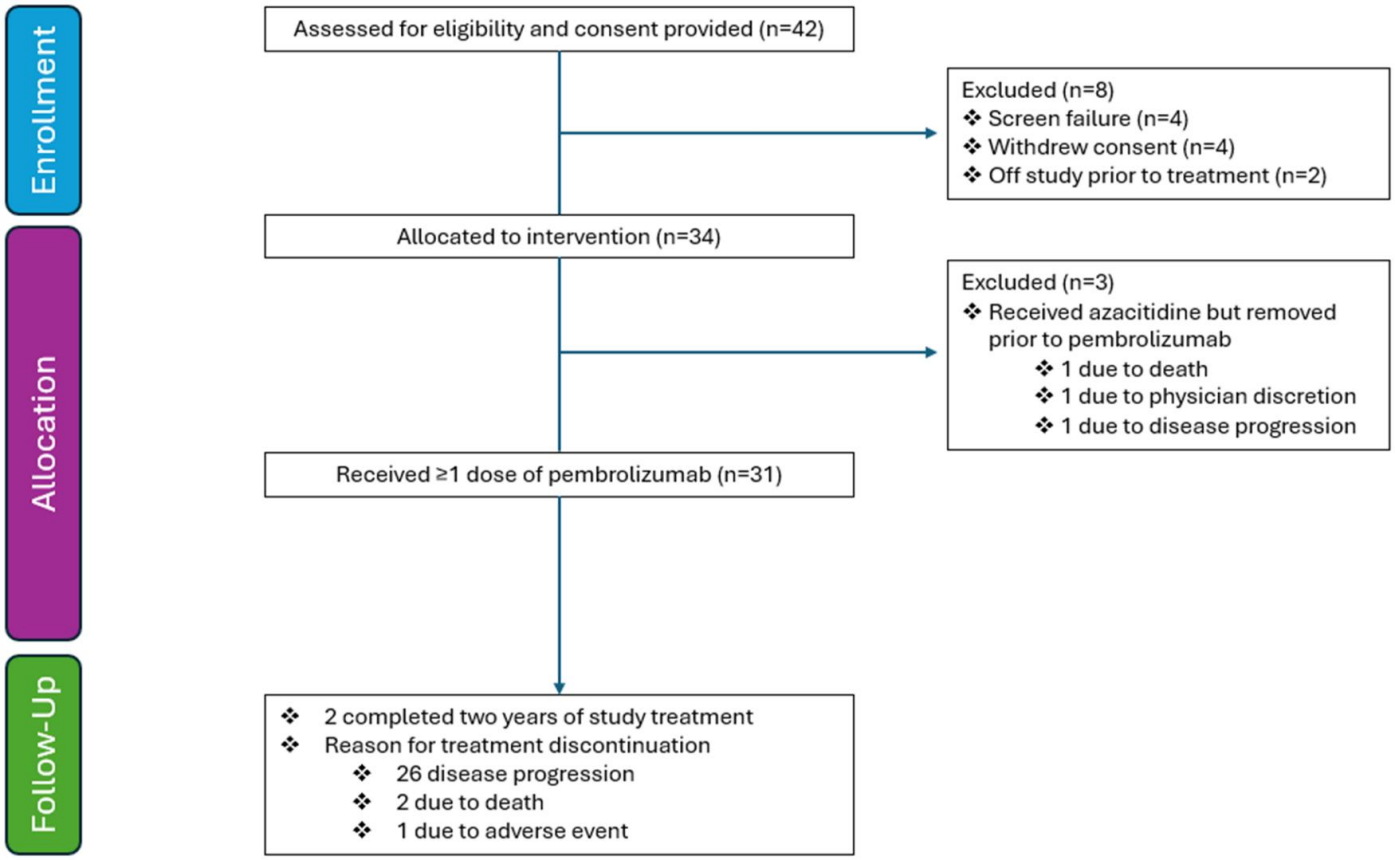
Patient flowchart. CONSORT diagram illustrates the inclusion of patients from enrollment to follow-up.

This was a single-arm, phase II study evaluating the safety and efficacy of azacitidine in combination with pembrolizumab in patients with locally advanced unresectable or metastatic PDAC who had progressed on a single line of 5-fluorouracil (5-FU)- or gemcitabine-based chemotherapy. The study aimed to enroll 31 evaluable patients (Figure 1).

The primary endpoint was progression-free survival (PFS defined as the time from treatment initiation to progression or death). Patients alive and progression-free at the time of analysis were censored at the date of their last assessment or follow-up if patient completed treatment. Radiographic assessments were performed every 8 weeks using CT or MRI, and CA 19-9 levels were monitored every 3 weeks. The statistical design was based on a null hypothesis that median PFS would be 2 months, with an alternative hypothesis of 4 months. A sample size of 31 evaluable patients provided 80% power to detect this difference at a 1-sided alpha of 0.05.

Secondary endpoints included overall response rate (ORR), duration of response (DOR), disease control rate (DCR), and overall survival (OS). OS was defined as the time from treatment initiation to death. Patients who were alive were censored at the time of their last follow-up. Analysis for primary and secondary endpoints are presented for patients who received at least one dose of azacitidine as well as those who received both agents. Patients were replaced if they did not receive at least a dose of pembrolizumab.

Descriptive statistics were used to summarize baseline data including median and ranges for continuous data and counts and percentages for categorical data. Time-to-event outcomes, including PFS and OS, were analyzed using Kaplan–Meier methods and reported along with their 95% corresponding intervals. ORR, DOR, and DCR were also summarized using frequencies.

**Table:** 

**PRIMARY ASSESSMENT METHOD**			
	Progression-free survival		
**Number of patients screened**	42		
**Number of patients enrolled**	36		
**Number of patients evaluable for toxicity**	34		
**Number of patients evaluated for efficacy**	31		
**Evaluation method**	RECIST 1.1		
**Response assessment**	***N* = 31**	**%**	
**CR**	0	0	
**PR**	3^a^	9.7%	
**SD**	8	25.8%	
**PD**	20	64.5%	
**(Median) duration assessment**	**Median**	**Day/week/month**	**95% CI**
**PFS**	1.51	Months	1.38, 3.42
**OS**	4.83	Months	4.27, 10.91

a One patient with a PR had an unconfirmed response; died of immune-related complications.

**Figure 2. | PFS and OS.**
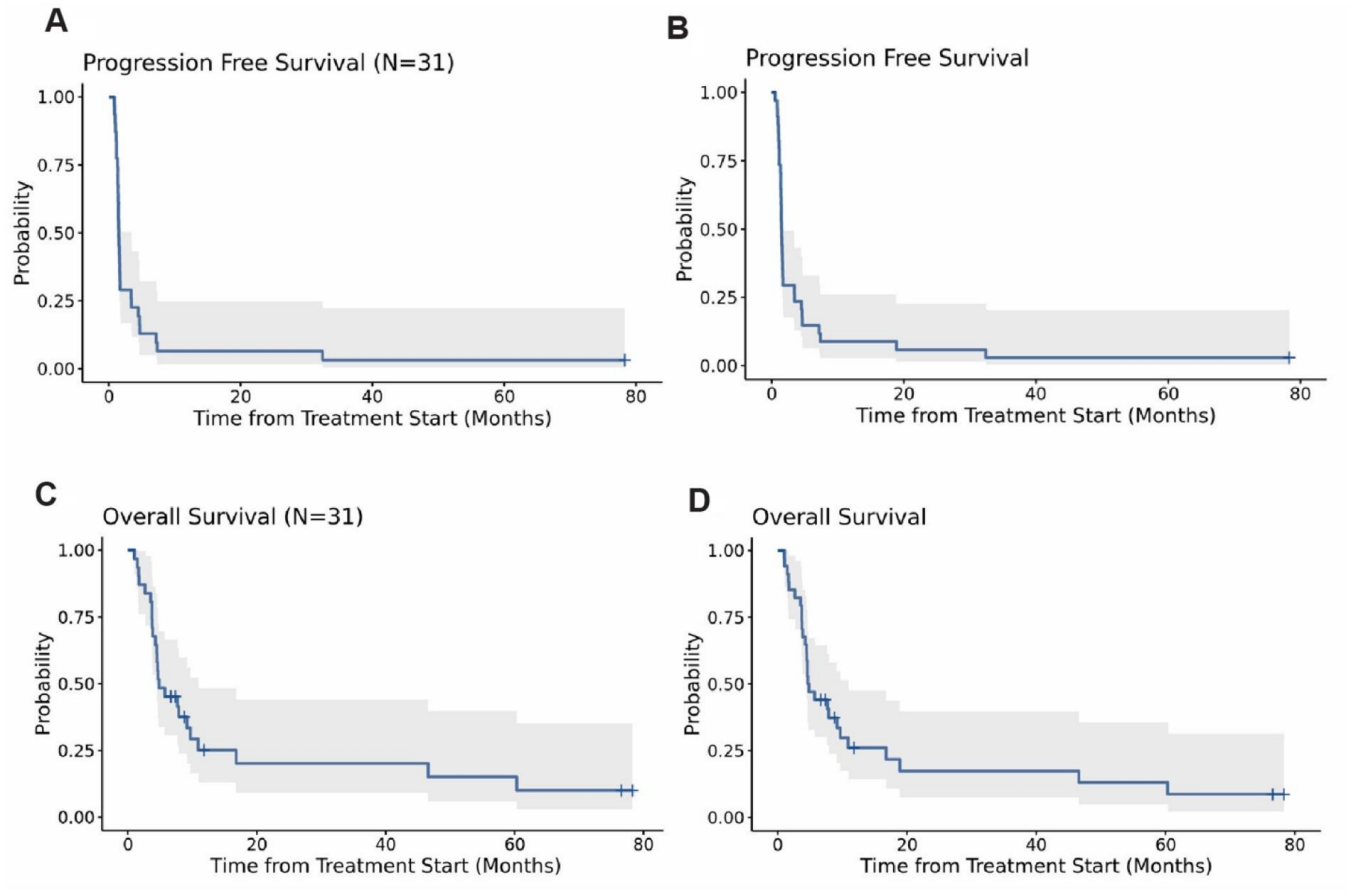
(A) Kaplan–Meier estimates of PFS for all 31 patients treated with at least one cycle of azacitidine and pembrolizumab and (B) all 34 patients evaluable for safety. (C) Kaplan–Meier estimates of OS for all 31 patients treated with at least one cycle of azacitidine and pembrolizumab and (D) all 34 patients evaluable for safety.

**Figure 3.**
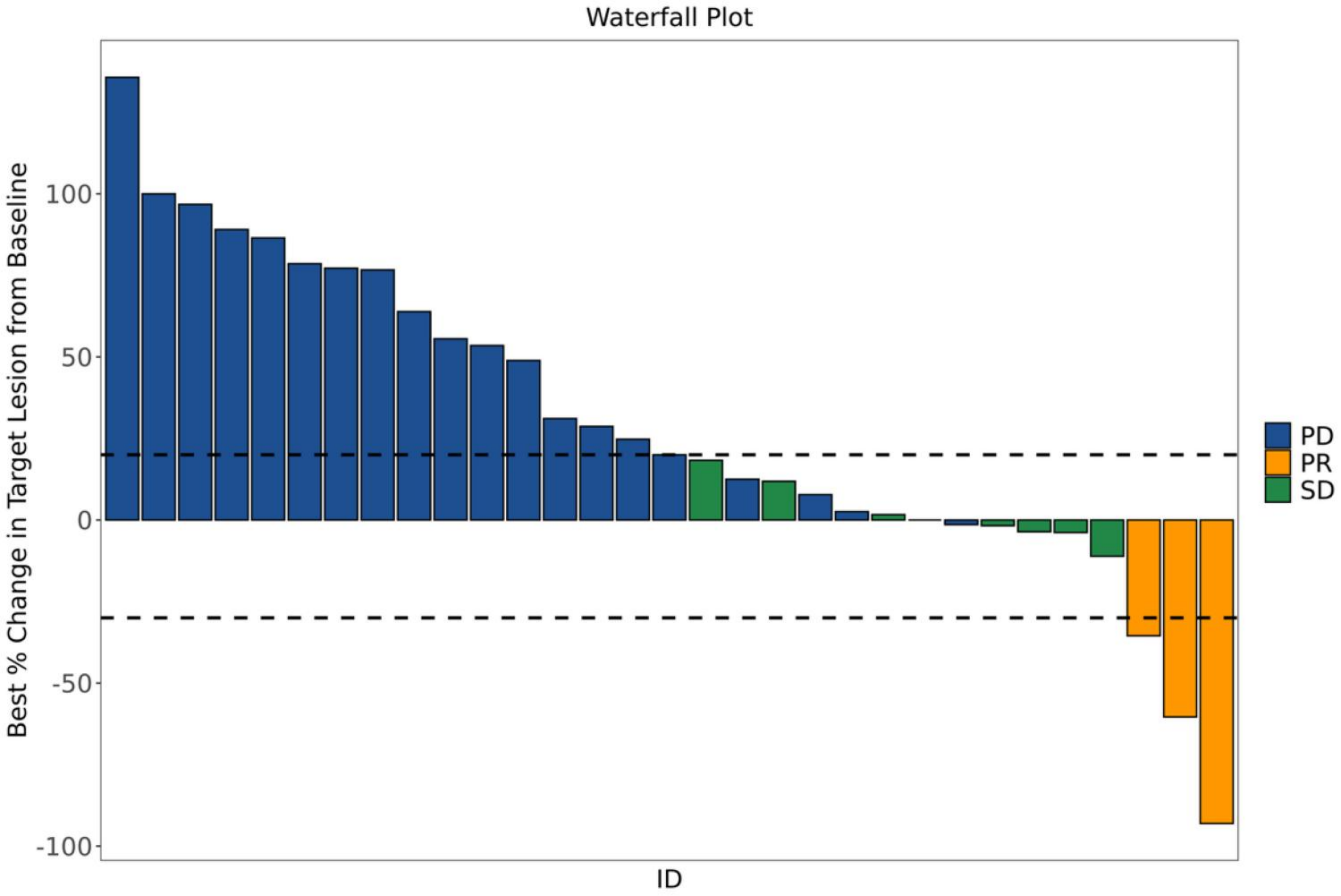
Waterfall plot for the best percentage change in target lesion size from baseline. Tumor measurements per RECIST 1.1 criteria were included for all evaluable patients (*n* = 31). Patients who had PD prior to the first scan were included as +20%.

**Figure 4.**
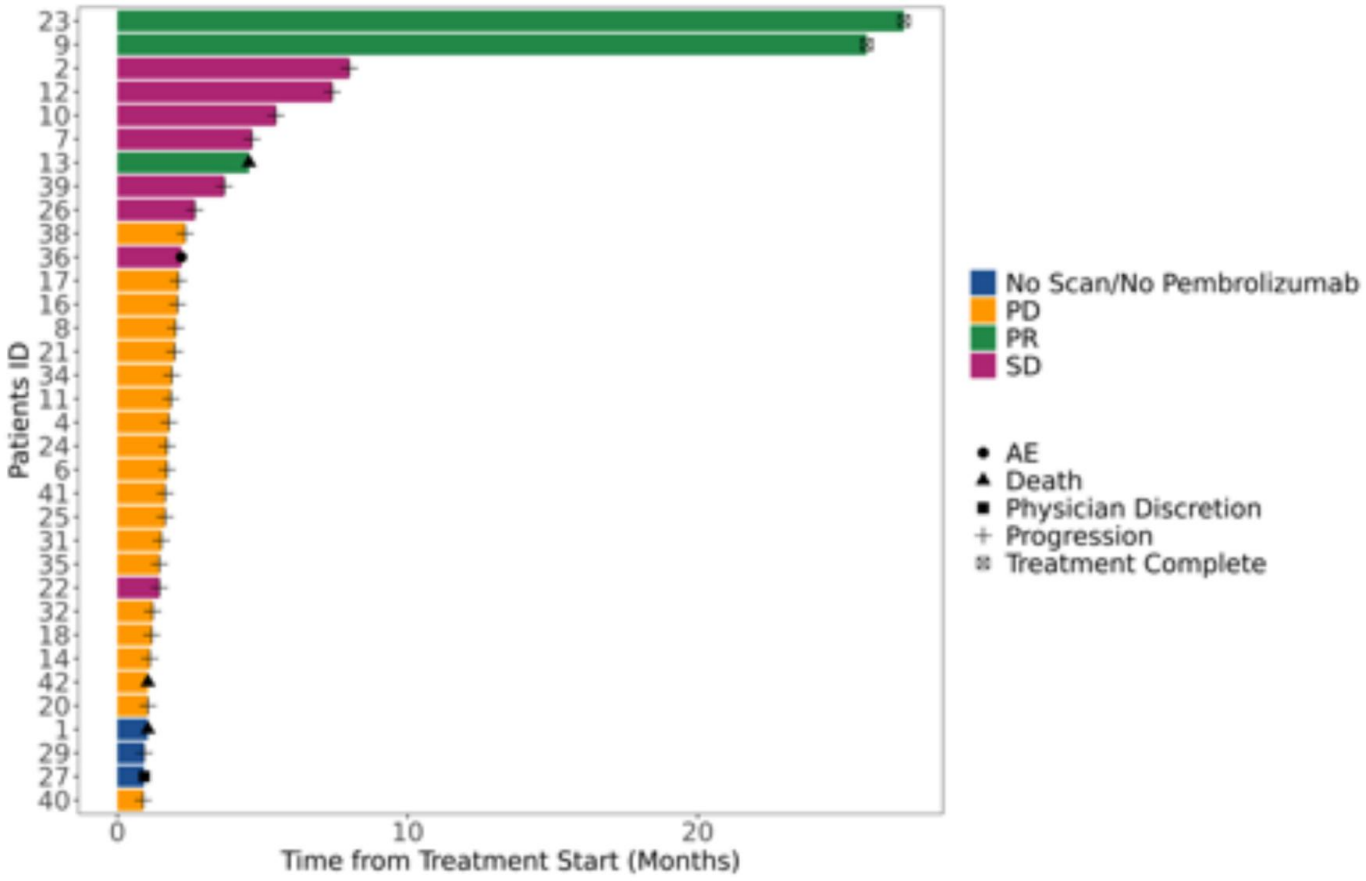
Best responses and duration of treatment. Two patients had a confirmed partial response; patient #13 had a PR that could not be confirmed as he died from an immune adverse event.

Thirty-four received at least one dose of azacitidine, and 31 received at least one dose of both agents. One patient remained alive and progression free at the time of the data lock in April 2025 with a follow-up of 78.4 months. Among patients who received ≥1 dose of azacitidine (safety group, Figure 2B and D), the median PFS was 1.49 months (95% CI: 1.38-1.74) and median OS was 4.75 months (95% CI: 4.47-10.91); for those who received ≥1 dose of pembrolizumab (efficacy group, Figure 2A and C), the median PFS was 1.51 months (95% CI: 1.38-3.42) and median OS was 4.83 (95% CI: 4.27-10.91) (Figure 2). Of the 31 patients in the efficacy group, 3 (9.7%) experienced a partial response (PR) and 8 (25.8%) SD resulting in a DCR of 35.5% (Figure 3). Of the 3 patients with a PR, 2 received 35 doses of pembrolizumab and continued receiving azacytidine, remaining on treatment over 2 years with ongoing response (Figure 4). The other patient’s PR was not confirmed after 4 weeks due to death following an immune-related encephalitis.

## Adverse events

Treatment-related adverse events (TRAEs) were reported in 20 patients (59%); the most common being diarrhea and fatigue. Grade ≥3 TRAEs occurred in 7 patients (21%) including 3 grade 5 events including immune-related encephalitis. Pembrolizumab was held for 10 (29.4%) patients, 8 of which were due to AEs.

**Table:** 

GENERAL TREATMENT-RELATED TOXICITY PROFILE			
adverse event	Grade 1-2 *N* (%)	Grade ≥3 *N* (%)	All grades *N* (%)
diarrhea	7 (20.6)	1 (2.9)	8 (23.5)
Fatigue	8 (23.5)	0	8 (23.5)
Anorexia	3 (8.8)	1 (2.9)	4 (11.8)
Thromboembolic events	0	2 (5.9)	2 (5.9)
Pembro-related	8 (23.5)	6 (17.6)	14 (41.2)

## Additional details of study design

### PD-L1 analysis

Method: Pre-treatment biopsies were stained for PD-L1 (Merck 22C3 antibody, QualTek). PD-L1 expression was scored as 0, +1, +2, +3 based on staining intensity and H-score calculated as the sum of the percentage of cells multiplied by the corresponding intensity score for each staining intensity.

**Figure 5.**
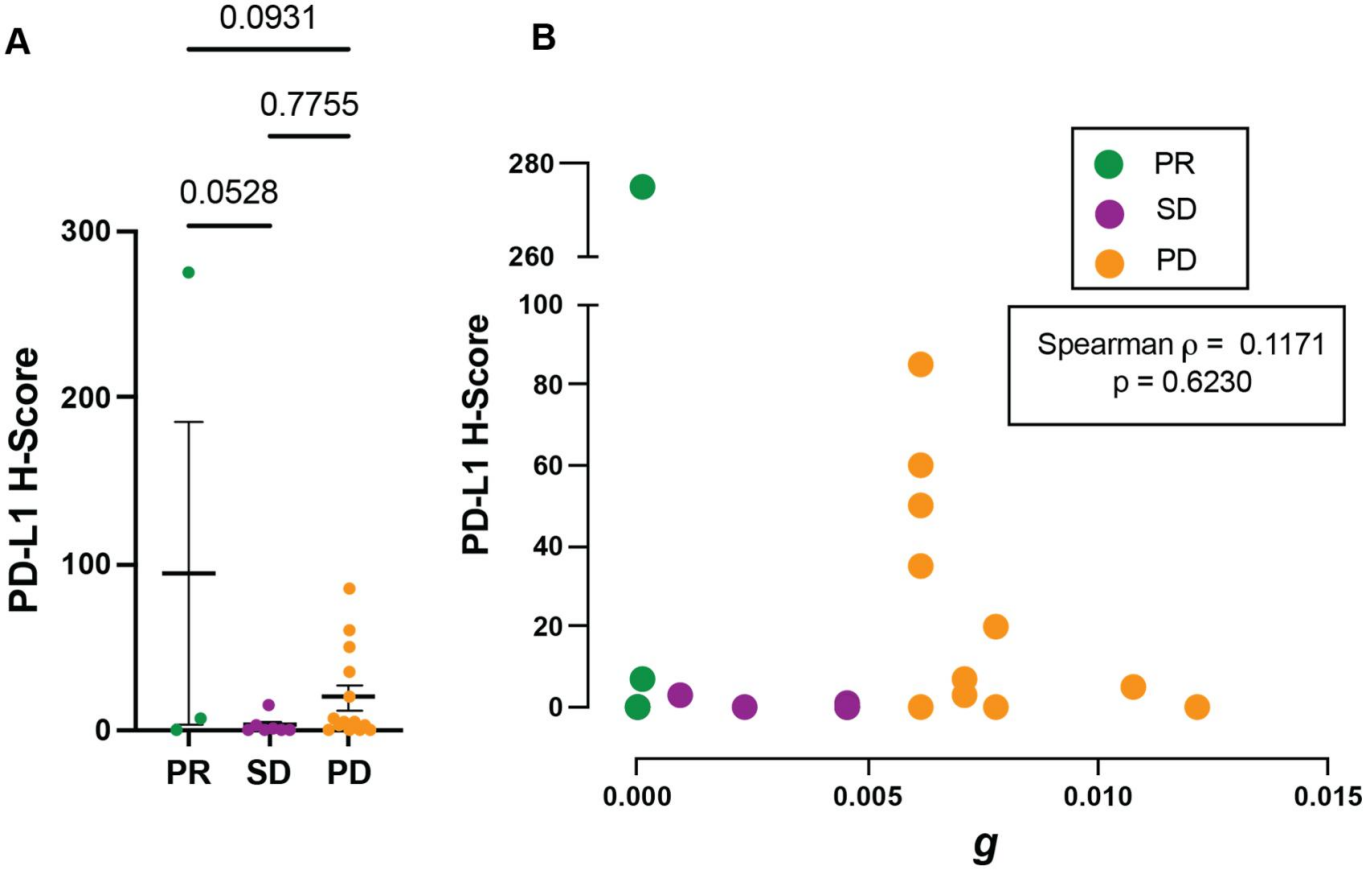
PD-L1 H-Score does not predict response or growth rate. (A) PD-L1 H-Scores were compared between patients that had best responses of PR (*n* = 3), SD (*n* = 7), and PD (*n* = 14). Error bars represent SEM. Significance was determined by one-way Anova with multiple comparisons, *P* values are displayed. (B) Growth rate (*g*) was calculated based on tumor imaging measurements as previously described.^1^ Correlation between PD-L1 H-score and *g* for each patient was assessed using Spearman’s test (*n* = 20).

Results: Of 31 evaluable patients, 24 had adequate tissue to generate the PD-L1 H score. There was no correlation between PD-L1 H-Score and best response to treatment (Figure 5A). An exponential tumor growth rate (***g***) was calculated for each patient based on a validated mathematical model of tumor growth kinetics using RECIST-directed imaging tumor measurements.^1^ We found no correlation between PD-L1 H-Score and tumor growth rate (Figure 5B).

### Germline and somatic testing

Methods: All patients underwent germline genetic testing and tumor next-generation sequencing (NGS) as part of clinical care.

Results: Tumor NGS was retrieved for 21 of 31 patients (Table 1). Of the 3 patients with disease response, 2 had tumors with a *KRAS* mutation. In one tumor, POLE L424I and F367V mutations, KRAS A146T mutation and TMB ∼ 237 Mut/Mb were found. The second patient had a somatic KRAS G12V mutation, while in the third, a BRCA1 Q1240* frameshift mutation was noted in the *KRAS* wild type setting. None of the patients were found to have microsatellite high or mismatch repair deficient disease.

**Table 1.** NGS mutation status in patients with response or stable disease.

Pt No.	**PR** ^†^ **(*n* = 3)**		SD (*n* = 8)
**9**	KRAS A146T, POLE L424I and F367V, APC PS943X and R1450X, PTEN E7X, SMAD4 R361H, TP53 R213X, ATM R250X, BCOR S214X, ERCC3 E55X, FAT1 R2597X, FAT1 E1147X, MLH1 R100X, MLH1 E512X, MSH6 E993X, NBN E254X, SMC3 R99X, TGFBR1 S241L, TNFAIP3 R87X, TP53BP1 E686X, TSC1 E813X	2	KRAS G12V TP53 R175H CDKN2A loss MYC E137Q
**13** ^†^	KRAS G12V, TP53 c.555 + 2T>G	7	KRAS G12D, SMAD V44Lfs
**23** ^#^	BRCA1 Q1240*, CDKN2A/B loss, NFE2L2 R34P, TERT promoter -124C>T	10	KRAS G12D, TP53 R196X, SMAD4 L121fs
		12^‡^	KRAS WT, TP53 R342
		36	KRAS G12D, SMAD4 E41*, TP53 N311fs*25

Does not include variants of uncertain significance or gene amplification.

† Includes patient #13 with an IRAE whose response could not be confirmed.

‡ Insufficient tissue for complete NGS report- KRAS wild type confirmed.

# This patient’s germline analysis indicated the presence of BRCA1 Q1240*.

### Tumor immune microenvironment

Methods: Immunohistochemistry (IHC) was performed on pre-treatment tumor biopsies with the following antibodies: anti-CD8a (1:200, CST, D8A8Y), anti-Granzyme B (1:200, CST, D6E9W), anti-Ki-67 (1:200, CST, 8D5), and anti-CD68 (1:400, CST, E3O7V). Quantification was performed in FIJI/ImageJ using the IHC Profiler plugin, with cell counts averaged across 3 high-power fields per section.

**Figure 6.**
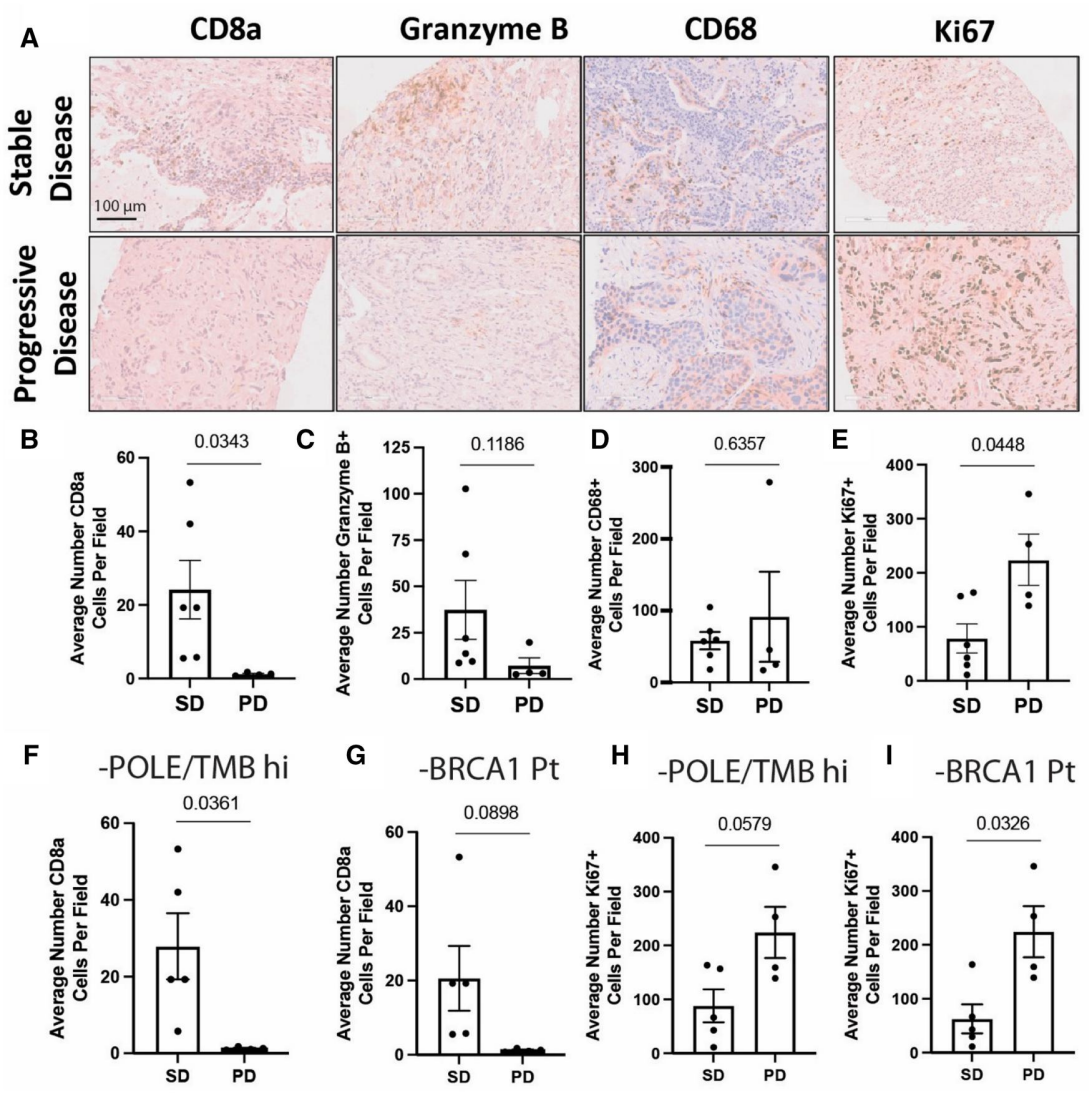
Increased baseline infiltrating CD8+ T cells and low Ki-67 are correlated with disease control after 8 weeks of AZA/pembro treatment. (A) Representative immunohistochemical (IHC) staining of immune (CD8a, Granzyme B, CD68) and proliferation markers (Ki67) in pre-treatment biopsy samples from patients with stable disease (SD) after 8 weeks of Aza/pembro treatment (top panels) and patients with progressive disease (PD) after 8 weeks of treatment. Quantification of CD8a (B), Granzyme B (C), CD68 (D), and Ki-67 (E) positive cells per high powered field in patients with SD (*n* = 6) and PD (*n* = 4) after 8 weeks of treatment with Aza/pembro. (F-I**)** Sensitivity analysis excluding subjects with POLE/TMB high and BRCA1 (G) mutations. Average CD8a cells per field subtracting the patients with POLE/TMB high (F) and BRCA1 mutations respectively. Average Ki67 cells per field subtracting the patients with POLE/TMB high (H) and BRCA1 (I) mutations. IHC staining was quantified using image J with the IHC plug-in. Staining was quantified in 4 separate fields for each patient. Bar graph shows means ± SEM. Significance assessed using Welches *t*-test, *P* values shown on graphs. Scale bar = 100 μm.

**Figure 7.**
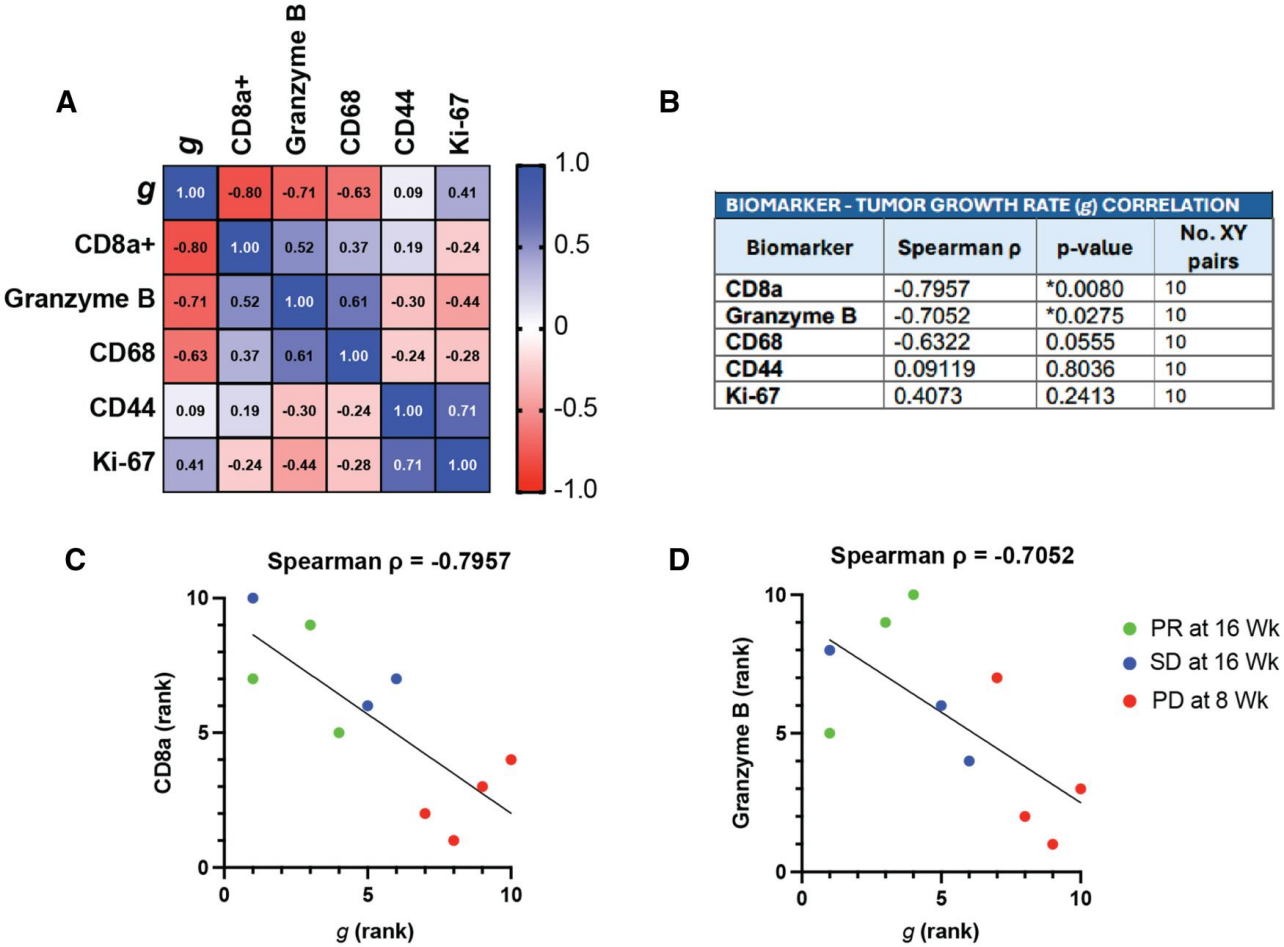
Higher baseline CD8a and Granzyme B tumor infiltration correlates with slower tumor growth rates in patients treated with Aza/pembro. (A) Spearman’s correlation matrix of exponential tumor growth rate (*g*) and baseline IHC biomarker quantification. (B) Summary table of Sperman’s correlation data and significance of *g* compared to each of the IHC tissue biomarkers. Correlation of ranked CD8a (C) and Granzyme B (D) staining and growth rate *g* by Spearmen’s rank highlighting patients progressing early at 8 weeks (PD), those with sustained stable disease at 16 weeks (SD) and those that achieved a partial response (PR) at 16 weeks.

Results: To explore biomarkers of response, IHC was performed on pre-treatment biopsies from patients with stable disease (SD) vs progressive disease (PD) at 8 weeks. Patients with SD had significantly higher densities of CD8^+^ T cells and a trend toward increased Granzyme B+ cells. CD68^+^ myeloid infiltration was similar between groups. Ki-67 staining was lower in patients with SD (Figure 6A-E). Two patients with RECIST-defined PR had mutations that could predict response to immunotherapy (POLE/TMB high and BRCA1). Sensitivity analyses removing these patients to assess potential outliers showed retention of trends in association (Figure 6F-I). Individual tumor *g* values were estimated and correlated with baseline CD8+ and granzyme B infiltration. Higher immune infiltration was correlated with lower ***g*** values (indicating slower tumor growth kinetics) (Figure 7).

## Discussion

This single-arm, phase II study evaluated azacitidine plus pembrolizumab in patients with locally advanced or metastatic PDAC after first-line therapy and completed accrual as planned. The regimen was well tolerated but demonstrated modest clinical activity, with a median PFS of 1.51 months, ORR of 9.7%, and a DCR of 35.5%, failing to meet the pre-specified efficacy threshold. One patient with a PR had pathogenic POLE mutations^2^ and high TMB,^3^ both of which are associated with clinical benefit to immune checkpoint inhibitors, and a second patient’s pathogenic BRCA mutation may have also sensitized the tumor to immune checkpoint blockade.^4^ These findings align with prior studies showing limited benefit of immune checkpoint inhibition in unselected patients with microsatellite stable PDAC.^5^^,^^6^ Whether azacitidine could have contributed to the >2 year clinical benefit from immunotherapy in the 2 patients with sensitizing mutations cannot be determined from the current study design.

Azacitidine was selected based on preclinical evidence that DNA hypomethylating agents enhance immune infiltration and sensitize tumors to PD-1 blockade.^7^ However, our findings suggest that epigenetic priming with azacitidine alone may be insufficient to overcome the profound immune resistance of PDAC, particularly in an unselected population. Combination strategies incorporating chemotherapy, multi-modal immune priming, or improved on-target drug delivery may be necessary to achieve meaningful clinical benefit.

Exploratory biomarker analyses on pre-treatment tumor samples suggest that pre-existing immune engagement may influence clinical outcomes. Higher baseline CD8^+^ T-cell infiltration correlated with treatment response, while baseline CD8+ T and Granzyme B+ cell infiltration inversely correlated with exponential growth rate (***g***) (Figure 7).^1^ In contrast, CD68+ myeloid cell density did not differ between responding and non-responding tumors. Further investigation on the effect of demethylation and PD-1 blockade may reveal selective effects on myeloid cell sub-populations. Overall, the data suggest that low baseline immune activation may contribute to treatment resistance. These findings align with our preclinical data in murine models of pancreatic cancer, demonstrating that decitabine plus PD-1 blockade increases T-cell infiltration.^7^^,^^8^ Prior studies demonstrating that DNA demethylation enhances T-cell activation and memory formation,^9^^,^^10^ raise the possibility that rational sequencing or combination strategies may improve efficacy.

We did not observe a correlation between classical or basal subtype, as defined by HMGA2/GATA6 staining,^11^ and treatment response. High Ki-67 expression was associated with progressive disease; however, this may reflect aggressive tumor biology rather than a treatment specific biomarker. PD-L1 expression, a common predictive biomarker in other malignancies, did not correlate with response or tumor growth rate (*g*) (Figure 5).

This study is limited by its small sample size, single-arm design, limited tissue for biomarker studies, including no paired pre- and post-treatment tissue and lack of a comparator arm. Nevertheless, it provides important insights into the immune biology of PDAC and highlights tumor immune contexture as a key determinant of response (and resistance) to epigenetic priming and checkpoint inhibition. This may inform future treatment strategies.

Given the modest clinical benefit observed, we do not recommend further development of azacitidine plus pembrolizumab in an unselected PDAC population. Future studies may consider alternative dosing or schedule of epigenetic agents, multi-modal priming strategies, incorporation of a chemotherapy backbone, or rational combinations that address immune evasion. Biomarker-driven strategies to enrich for immune-inflamed tumors may be necessary to unlock the potential of these therapies. These findings underscore the need for innovative, biology-driven approaches to improve outcomes in this treatment-refractory disease.

## Data Availability

Data will be made available subject to a reasonable request.
